# Bis[(1S*,2S*)-*trans*-1,2-bis­(diphenyl­phosphin­oxy)cyclo­hexa­ne]chlorido­ruthenium(II) trifluoro­methane­sulfonate dichloro­methane disolvate

**DOI:** 10.1107/S1600536809023034

**Published:** 2009-06-20

**Authors:** George R. Clark, Cornelis Lensink, Angela T. Slade, L. James Wright

**Affiliations:** a Department of Chemistry, The University of Auckland, Private Bag 92019, Auckland, New Zealand; b Industrial Research Limited, PO Box 31-310, Lower Hutt, New Zealand

## Abstract

The crystal structure of a racemic mixture of the title ruthenium(II) complex, [RuCl(C_30_H_30_O_2_P_2_)_2_]CF_3_SO_3_·2CH_2_Cl_2_, reveals that the coordination geometry about the coordinatively unsaturated metal centre is approximately trigonal-pyramidal, with the chlorine atom occupying one of the equatorial positions. The axial Ru—P bonds are longer than the equatorial Ru—P bonds and there is an acute P—Ru—P angle.

## Related literature

For the syntheses and properties of chiral asymmetric hydrogenation catalysts, see: Knowles & Noyori (2007 ▶); Zhang *et al.* (2007 ▶); Zhang (2004 ▶). For the syntheses and properties of chiral diphosphinite complexes, see: Au-Yeung & Chan (2004 ▶); Falshaw *et al.* (2007 ▶); Clark *et al.* (2009 ▶). For a decription of the Cambridge Structural Database, see: Allen (2002 ▶).
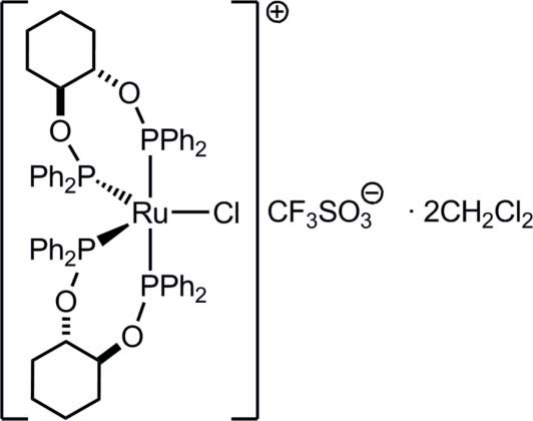


## Experimental

### 

#### Crystal data

[RuCl(C_30_H_30_O_2_P_2_)_2_]CF_3_SO_3_·2CH_2_Cl_2_
*M* *_r_* = 1424.40
Orthorhombic, 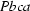

*a* = 16.7887 (5) Å
*b* = 22.9766 (6) Å
*c* = 32.6782 (9) Å
*V* = 12605.5 (6) Å^3^
*Z* = 8
Mo *K*α radiation
μ = 0.66 mm^−1^
*T* = 85 K
0.32 × 0.18 × 0.10 mm

#### Data collection

Siemens SMART CCD diffractometer
Absorption correction: multi-scan (*SADABS*; Sheldrick, 1996 ▶) *T*_min_ = 0.808, *T*_max_ = 0.930
70582 measured reflections
12041 independent reflections
8363 reflections with *I* > 2σ(*I*)
*R*_int_ = 0.049

#### Refinement

*R*[*F* ^2^ > 2σ(*F* ^2^)] = 0.059
*wR*(*F* ^2^) = 0.136
*S* = 1.06
12041 reflections
757 parameters
H-atom parameters constrained
Δρ_max_ = 1.10 e Å^−3^
Δρ_min_ = −1.16 e Å^−3^

### 

Data collection: *SMART* (Siemens, 1995 ▶); cell refinement: *SAINT* (Siemens, 1995 ▶); data reduction: *SAINT*; program(s) used to solve structure: *SHELXS97* (Sheldrick, 2008 ▶); program(s) used to refine structure: *SHELXL97* (Sheldrick, 2008 ▶); molecular graphics: *ORTEP*-III (Burnett & Johnson, 1996 ▶); software used to prepare material for publication: *SHELXTL* (Sheldrick, 2008 ▶).

## Supplementary Material

Crystal structure: contains datablocks global, I. DOI: 10.1107/S1600536809023034/lh2843sup1.cif

Structure factors: contains datablocks I. DOI: 10.1107/S1600536809023034/lh2843Isup2.hkl

Additional supplementary materials:  crystallographic information; 3D view; checkCIF report

## Figures and Tables

**Table:** 

Ru—P2	2.2237 (13)
Ru—P3	2.2430 (13)
Ru—Cl1	2.3838 (13)
Ru—P1	2.3935 (12)
Ru—P4	2.4170 (13)

**Table:** 

P2—Ru—P3	87.81 (5)
P2—Ru—Cl1	131.42 (5)
P3—Ru—Cl1	140.73 (5)
P2—Ru—P1	89.44 (4)
P3—Ru—P1	99.68 (4)
Cl1—Ru—P1	84.49 (4)
P2—Ru—P4	99.48 (4)
P3—Ru—P4	89.39 (4)
Cl1—Ru—P4	83.10 (4)
P1—Ru—P4	167.55 (4)

**Fig. 1.**
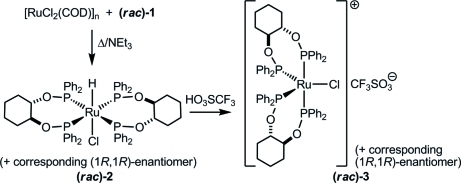
Reaction scheme.

**Fig. 2.**
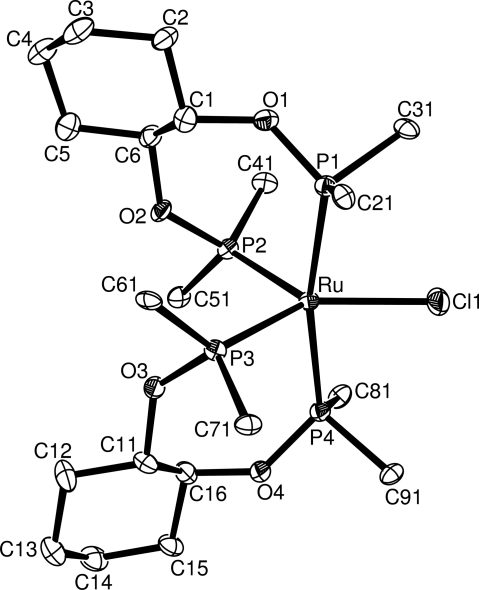
The molecular structure of the cation [RuCl{(1S,2S)-trans-(OPPh2)2(C6H10)}2] O3SCF3 of (rac)-3 showing 50% probability displacement ellipsoids for non-hydrogen atoms (Burnett & Johnson, 1996). For clarity, only the ipso carbon atoms of the phenyl rings are depicted.

## supplementary crystallographic information

### Comment

The development and study of new asymmetric hydrogenation catalysts continues to be a very active area of research (Knowles & Noyori, 2007). Reasons for this interest include the commercial importance of producing enantiomerically pure organic materials (especially for the pharmaceutical industry) and the fact that successful catalysts tend to be substrate-specific rather than being generally useful for a wide range of prochiral substrates (Zhang *et al.*, 2007, Zhang, 2004). Many of the successful catalysts that have been developed contain chiral phosphane or phosphinite ligands (Au-Yeung & Chan, 2004). In our recent studies in this area we have synthesized and studied a range of new chiral ruthenium complexes that are potential asymmetric hydrogenation catalysts (Falshaw *et al.*, 2007, Clark *et al.*, 2009). These complexes all contain chiral diphosphinite ligands that have either *chiro*-inositol or cyclohexane backbones. During these investigations we prepared a racemic mixture of the cationic, chiral ruthenium complexes [RuCl{(1*S*,2*S*)-*trans*-(OPPh_2_)_2_(C_6_H_10_)}_2_]O_3_SCF_3_ and [RuCl{(1*R*,2*R*) -*trans*-(OPPh_2_)_2_(C_6_H_10_)}_2_]O_3_SCF_3_((*rac*)-3) through treatment of a racemic mixture of the corresponding hydride complexes ((*rac*)-2) with triflic acid (see Figure 1). (*rac*)-2, in turn, was prepared by heating a solution of [RuCl_2_(COD)]_n_ with NEt_3_ and a racemic mixture of the diphosphinite ligands (1*R*,2*R*)-1,2-*trans*-bis-(*O*-diphenylphosphino)cyclohexane and (1*S*,2*S*)-1,2-*trans*-bis-(*O*-diphenylphosphino)cyclohexane ((*rac*)-1). We now report the details of the structure of (*rac*)-3 which crystallizes with four molecules of each enantiomer in the unit cell. The bond lengths and angles for each enantiomer are crystallographically identical and the structure of [RuCl{(1*S*,2*S*)-*trans*-(OPPh_2_)_2_(C_6_H_10_)}_2_]O_3_SCF_3_ only is depicted in Figure 2. The geometry about the ruthenium(II) centre in this coordinatively unsaturated complex is approximately trigonal bipyramidal with chloride occupying one of the equatorial positions. It is noteworthy that the isomer of [RuCl{(1*S*,2*S*)-*trans*-(OPPh_2_)_2_(C_6_H_10_)} _2_]O_3_SCF_3_ that has the opposite configuration at the metal centre was not present in the crystal. As expected, the two phosphorus atoms in the axial positions (P1 and P4; P1—Ru—P4 = 167.55 (4)°) form slightly longer bonds to ruthenium (Ru—P1 = 2.3935 (13), Ru—P4 = 2.4170 (13) Å) than the two phosphorus atoms (P2 and P3) that are in the equatorial positions (Ru—P2 = 2.2237 (13), Ru—P3 = 2.2430 (13) Å). However, all Ru—P distances fall within the normal range for compounds of this type [Cambridge Structure Database Version 5.30; Allen (2002); average Ru-P(OR)Ph_2_ distance = 2.288Å (SD = 0.042Å]. Similarly, the Ru—Cl distance (2.3838 (13) Å) is normal. The P2—Ru—P3 angle is small at 87.81 (5)°. The crystals also contain two dichloromethane molecules of crystallization per molecule of complex.

### Experimental

Synthesis of a racemic mixture of chlorobis{(1*S*,2*S*)-1,2-*trans*-bis-(*O*-diphenylphosphino)cyclohexane}ruthenium(II) trifluoromethanesulfonate and chlorobis{(1*R*,2*R*)-1,2-*trans*-bis-(*O*-diphenylphosphino)cyclohexane}ruthenium(II) trifluoromethanesulfonate (*rac*-3). Triflic acid (0.049 ml, 0.56 mmol) was added under nitrogen to a racemic mixture of RuHCl{(1*S*,2*S*)-*trans*-(OPPh_2_)_2_(C_6_H_10_)}_2_ and RuHCl{(1*R*,2*R*)- *trans*-(OPPh_2_)_2_(C_6_H_10_)}_2_ (0.21 g, 0.19 mmol) in THF (10 ml) and toluene (1 ml). The solution was stirred for 15 minutes at R.T. and the solvents were removed under reduced pressure to give a red product that was recrystallized from dichloromethane/hexane. MS (*m/z*): Calcd for C_60_H_60_^35^ ClO_4_P_4_^102^Ru (*M*^+^) 1105.21741 *m/z*. Found: 1105.21644. ^1^H NMR (CDCl_3_, δ): 0.74–2.40 (m, 16H, C*H*_2_), 3.70–4.90 (m, 4H, C*H*), 6.58–7.90 (m, 40H, *Ph*). ^13^C NMR (CDCl_3_, δ): 22.4 (*C*H_2_), 23.3 (*C*H_2_), 31.7 (*C*H_2_), 32.8 (*C*H_2_), 77.9 (*C*H), 83.1 (*C*H), 126.0–136.0 (multiple signals, Ph). ^31^P{^1^H} NMR (CDCl_3_,δ): 126.18 (t, ^2^J_PP_ = 29.6 Hz), 157.19 (t, ^2^J_PP_ = 29.6 Hz).

### Refinement

Hydrogen atoms were placed in calculated positions and refined using the riding model [C—H 0.93–0.97 Å), with *U*_iso_(H) = 1.2 or 1.5 times *U*_eq_(C). At the completion of refinement, the second parameter of WGHT (55.0) is quite large, possibly as a consequence of the generally weak nature of the X-ray intensity data.

### Crystal data

**Table:**

[RuCl(C_30_H_30_O_2_P_2_)_2_]CF_3_SO_3_·2CH_2_Cl_2_	*F*(000) = 5840
*M**_r_* = 1424.40	*D*_x_ = 1.501 Mg m^−^^3^
Orthorhombic, *P**b**c**a*	Mo *K*α radiation, λ = 0.71073 Å
Hall symbol: -p 2ac 2ab	Cell parameters from 8192 reflections
*a* = 16.7887 (5) Å	θ = 1.6–25.8°
*b* = 22.9766 (6) Å	µ = 0.66 mm^−^^1^
*c* = 32.6782 (9) Å	*T* = 85 K
*V* = 12605.5 (6) Å^3^	Needle, orange
*Z* = 8	0.32 × 0.18 × 0.10 mm

### Data collection

**Table:**

Siemens SMART CCD diffractometer	12041 independent reflections
Radiation source: fine-focus sealed tube	8363 reflections with *I* > 2σ(*I*)
graphite	*R*_int_ = 0.049
Area detector ω scans	θ_max_ = 25.8°, θ_min_ = 1.6°
Absorption correction: multi-scan *SADABS*; Sheldrick, 1996	*h* = 0→20
*T*_min_ = 0.808, *T*_max_ = 0.930	*k* = 0→28
70582 measured reflections	*l* = 0→39

### Refinement

**Table:**

Refinement on *F*^2^	Primary atom site location: structure-invariant direct methods
Least-squares matrix: full	Secondary atom site location: difference Fourier map
*R*[*F*^2^ > 2σ(*F*^2^)] = 0.059	Hydrogen site location: inferred from neighbouring sites
*wR*(*F*^2^) = 0.136	H-atom parameters constrained
*S* = 1.06	*w* = 1/[σ^2^(*F*_o_^2^) + (0.0418*P*)^2^ + 54.6685*P*] where *P* = (*F*_o_^2^ + 2*F*_c_^2^)/3
12041 reflections	(Δ/σ)_max_ = 0.001
757 parameters	Δρ_max_ = 1.10 e Å^−^^3^
0 restraints	Δρ_min_ = −1.16 e Å^−^^3^

### Special details

**Table:**

Geometry. All e.s.d.'s (except the e.s.d. in the dihedral angle between two l.s. planes) are estimated using the full covariance matrix. The cell e.s.d.'s are taken into account individually in the estimation of e.s.d.'s in distances, angles and torsion angles; correlations between e.s.d.'s in cell parameters are only used when they are defined by crystal symmetry. An approximate (isotropic) treatment of cell e.s.d.'s is used for estimating e.s.d.'s involving l.s. planes.
Refinement. Refinement of *F*^2^ against ALL reflections. The weighted *R*-factor *wR* and goodness of fit *S* are based on *F*^2^, conventional *R*-factors *R* are based on *F*, with *F* set to zero for negative *F*^2^. The threshold expression of *F*^2^ > σ(*F*^2^) is used only for calculating *R*-factors(gt) *etc*. and is not relevant to the choice of reflections for refinement. *R*-factors based on *F*^2^ are statistically about twice as large as those based on *F*, and *R*- factors based on ALL data will be even larger.

### Fractional atomic coordinates and isotropic or equivalent isotropic displacement parameters (Å^2^)

**Table:**

	*x*	*y*	*z*	*U*_iso_*/*U*_eq_	
Ru	0.36096 (2)	0.821139 (16)	0.162484 (11)	0.01309 (10)	
Cl1	0.34785 (8)	0.81826 (6)	0.23509 (4)	0.0285 (3)	
Cl2	0.78357 (12)	0.81608 (10)	0.12744 (9)	0.0868 (8)	
Cl3	0.94715 (12)	0.78269 (9)	0.14459 (7)	0.0707 (6)	
Cl4	0.84460 (11)	0.69420 (9)	0.05105 (5)	0.0596 (5)	
Cl5	0.90640 (12)	0.59698 (9)	0.09840 (8)	0.0778 (7)	
S	0.64455 (8)	0.55087 (6)	0.10378 (4)	0.0245 (3)	
P1	0.41043 (7)	0.72403 (5)	0.17001 (4)	0.0154 (3)	
P2	0.28069 (7)	0.79050 (5)	0.11283 (4)	0.0149 (3)	
P3	0.44373 (7)	0.85361 (5)	0.11368 (4)	0.0146 (3)	
P4	0.31043 (7)	0.91891 (5)	0.17092 (4)	0.0155 (3)	
F1	0.7117 (2)	0.49198 (16)	0.04455 (11)	0.0509 (10)	
F2	0.6522 (2)	0.43894 (14)	0.08938 (10)	0.0469 (9)	
F3	0.5832 (2)	0.48723 (15)	0.04537 (11)	0.0473 (9)	
O1	0.39459 (19)	0.67565 (14)	0.13525 (10)	0.0182 (7)	
O2	0.32316 (18)	0.75725 (13)	0.07524 (10)	0.0169 (7)	
O3	0.40149 (19)	0.89216 (13)	0.07866 (9)	0.0166 (7)	
O4	0.3252 (2)	0.96987 (14)	0.13792 (10)	0.0197 (7)	
O5	0.6393 (2)	0.60028 (15)	0.07688 (10)	0.0266 (8)	
O6	0.5733 (2)	0.53826 (18)	0.12712 (11)	0.0346 (10)	
O7	0.7175 (2)	0.54643 (15)	0.12665 (11)	0.0251 (8)	
C1	0.4121 (3)	0.6764 (2)	0.09159 (14)	0.0189 (10)	
H1	0.4572	0.7023	0.0858	0.023*	
C2	0.4320 (3)	0.6141 (2)	0.07991 (15)	0.0216 (11)	
H2A	0.3877	0.5888	0.0869	0.026*	
H2B	0.4783	0.6011	0.0951	0.026*	
C3	0.4486 (3)	0.6101 (2)	0.03408 (16)	0.0265 (12)	
H3A	0.4942	0.6342	0.0274	0.032*	
H3B	0.4616	0.5702	0.0270	0.032*	
C4	0.3771 (3)	0.6298 (2)	0.00926 (17)	0.0269 (12)	
H4A	0.3339	0.6022	0.0129	0.032*	
H4B	0.3910	0.6304	−0.0195	0.032*	
C5	0.3492 (3)	0.6903 (2)	0.02230 (15)	0.0225 (11)	
H5A	0.2988	0.6988	0.0091	0.027*	
H5B	0.3877	0.7189	0.0130	0.027*	
C6	0.3390 (3)	0.6960 (2)	0.06875 (15)	0.0188 (11)	
H6	0.2930	0.6730	0.0776	0.023*	
C11	0.3956 (3)	0.9549 (2)	0.07476 (15)	0.0191 (11)	
H11	0.4409	0.9731	0.0887	0.023*	
C12	0.4000 (3)	0.9688 (2)	0.02941 (15)	0.0276 (12)	
H12A	0.3582	0.9480	0.0150	0.033*	
H12B	0.4509	0.9562	0.0186	0.033*	
C13	0.3902 (3)	1.0340 (2)	0.02276 (17)	0.0305 (13)	
H13A	0.4341	1.0545	0.0355	0.037*	
H13B	0.3911	1.0425	−0.0063	0.037*	
C14	0.3118 (3)	1.0549 (2)	0.04109 (16)	0.0280 (12)	
H14A	0.3071	1.0966	0.0374	0.034*	
H14B	0.2678	1.0364	0.0270	0.034*	
C15	0.3077 (3)	1.0405 (2)	0.08646 (15)	0.0216 (11)	
H15A	0.2564	1.0527	0.0972	0.026*	
H15B	0.3487	1.0620	0.1009	0.026*	
C16	0.3188 (3)	0.9755 (2)	0.09446 (15)	0.0193 (11)	
H16	0.2733	0.9533	0.0841	0.023*	
C21	0.5168 (3)	0.7227 (2)	0.18084 (14)	0.0157 (10)	
C22	0.5640 (3)	0.6762 (2)	0.16839 (15)	0.0212 (11)	
H22	0.5415	0.6455	0.1540	0.025*	
C23	0.6445 (3)	0.6759 (2)	0.17755 (16)	0.0244 (11)	
H23	0.6760	0.6448	0.1690	0.029*	
C24	0.6787 (3)	0.7212 (2)	0.19929 (16)	0.0233 (11)	
H24	0.7327	0.7206	0.2055	0.028*	
C25	0.6320 (3)	0.7673 (2)	0.21173 (16)	0.0228 (11)	
H25	0.6549	0.7979	0.2261	0.027*	
C26	0.5511 (3)	0.7682 (2)	0.20297 (15)	0.0200 (11)	
H26	0.5198	0.7992	0.2118	0.024*	
C31	0.3744 (3)	0.6792 (2)	0.21264 (14)	0.0181 (10)	
C32	0.3181 (3)	0.6356 (2)	0.20568 (16)	0.0228 (11)	
H32	0.2978	0.6299	0.1795	0.027*	
C33	0.2924 (3)	0.6005 (2)	0.23776 (18)	0.0300 (13)	
H33	0.2555	0.5712	0.2329	0.036*	
C34	0.3217 (3)	0.6093 (2)	0.27698 (18)	0.0317 (14)	
H34	0.3032	0.5866	0.2985	0.038*	
C35	0.3783 (3)	0.6518 (2)	0.28402 (17)	0.0276 (13)	
H35	0.3989	0.6570	0.3102	0.033*	
C36	0.4044 (3)	0.6869 (2)	0.25212 (15)	0.0203 (11)	
H36	0.4422	0.7156	0.2571	0.024*	
C41	0.2037 (3)	0.7407 (2)	0.13072 (15)	0.0179 (10)	
C42	0.1524 (3)	0.7164 (2)	0.10137 (16)	0.0216 (11)	
H42	0.1591	0.7254	0.0738	0.026*	
C43	0.0919 (3)	0.6794 (2)	0.11334 (16)	0.0240 (11)	
H43	0.0581	0.6634	0.0938	0.029*	
C44	0.0816 (3)	0.6659 (2)	0.15415 (17)	0.0277 (12)	
H44	0.0410	0.6406	0.1619	0.033*	
C45	0.1309 (3)	0.6896 (2)	0.18354 (17)	0.0241 (11)	
H45	0.1233	0.6806	0.2110	0.029*	
C46	0.1924 (3)	0.7271 (2)	0.17193 (15)	0.0183 (11)	
H46	0.2257	0.7431	0.1917	0.022*	
C51	0.2204 (3)	0.8402 (2)	0.08235 (14)	0.0175 (10)	
C52	0.2425 (3)	0.8541 (2)	0.04189 (15)	0.0204 (11)	
H52	0.2892	0.8389	0.0309	0.024*	
C53	0.1951 (3)	0.8901 (2)	0.01849 (16)	0.0279 (13)	
H53	0.2103	0.8992	−0.0081	0.033*	
C54	0.1253 (3)	0.9129 (2)	0.03420 (17)	0.0301 (13)	
H54	0.0943	0.9379	0.0185	0.036*	
C55	0.1021 (3)	0.8983 (2)	0.07345 (17)	0.0284 (13)	
H55	0.0549	0.9132	0.0841	0.034*	
C56	0.1484 (3)	0.8617 (2)	0.09692 (16)	0.0217 (11)	
H56	0.1312	0.8512	0.1230	0.026*	
C61	0.5054 (3)	0.80913 (19)	0.07959 (15)	0.0162 (10)	
C62	0.5730 (3)	0.7816 (2)	0.09426 (16)	0.0206 (11)	
H62	0.5861	0.7846	0.1218	0.025*	
C63	0.6215 (3)	0.7497 (2)	0.06817 (17)	0.0248 (12)	
H63	0.6662	0.7307	0.0784	0.030*	
C64	0.6033 (3)	0.7460 (2)	0.02706 (17)	0.0294 (13)	
H64	0.6355	0.7244	0.0096	0.035*	
C65	0.5375 (3)	0.7744 (2)	0.01185 (17)	0.0271 (12)	
H65	0.5260	0.7726	−0.0160	0.033*	
C66	0.4880 (3)	0.8058 (2)	0.03791 (15)	0.0219 (11)	
H66	0.4433	0.8246	0.0275	0.026*	
C71	0.5219 (3)	0.8987 (2)	0.13560 (15)	0.0178 (10)	
C72	0.5796 (3)	0.9220 (2)	0.10925 (16)	0.0216 (11)	
H72	0.5764	0.9149	0.0813	0.026*	
C73	0.6409 (3)	0.9551 (2)	0.12447 (17)	0.0287 (12)	
H73	0.6787	0.9708	0.1068	0.034*	
C74	0.6463 (3)	0.9652 (2)	0.16619 (17)	0.0290 (12)	
H74	0.6884	0.9871	0.1764	0.035*	
C75	0.5903 (3)	0.9432 (2)	0.19249 (16)	0.0257 (12)	
H75	0.5944	0.9505	0.2204	0.031*	
C76	0.5274 (3)	0.9100 (2)	0.17760 (15)	0.0207 (11)	
H76	0.4893	0.8953	0.1955	0.025*	
C81	0.2040 (3)	0.9219 (2)	0.18108 (15)	0.0196 (11)	
C82	0.1647 (3)	0.8756 (2)	0.19968 (15)	0.0222 (11)	
H82	0.1933	0.8430	0.2079	0.027*	
C83	0.0830 (3)	0.8777 (2)	0.20608 (16)	0.0280 (12)	
H83	0.0570	0.8466	0.2185	0.034*	
C84	0.0406 (3)	0.9262 (3)	0.19393 (18)	0.0357 (14)	
H84	−0.0142	0.9274	0.1978	0.043*	
C85	0.0791 (3)	0.9729 (3)	0.17612 (18)	0.0346 (14)	
H85	0.0502	1.0057	0.1685	0.041*	
C86	0.1607 (3)	0.9710 (2)	0.16954 (17)	0.0280 (12)	
H86	0.1864	1.0025	0.1574	0.034*	
C91	0.3491 (3)	0.9619 (2)	0.21382 (14)	0.0173 (10)	
C92	0.4063 (3)	1.0052 (2)	0.20654 (16)	0.0214 (11)	
H92	0.4265	1.0102	0.1803	0.026*	
C93	0.4329 (3)	1.0405 (2)	0.23789 (16)	0.0260 (12)	
H93	0.4709	1.0690	0.2326	0.031*	
C94	0.4036 (3)	1.0337 (2)	0.27663 (16)	0.0248 (12)	
H94	0.4215	1.0576	0.2977	0.030*	
C95	0.3467 (3)	0.9909 (2)	0.28454 (15)	0.0217 (11)	
H95	0.3274	0.9859	0.3110	0.026*	
C96	0.3191 (3)	0.9560 (2)	0.25330 (14)	0.0182 (10)	
H96	0.2801	0.9283	0.2586	0.022*	
C97	0.6475 (4)	0.4893 (3)	0.06932 (17)	0.0368 (14)	
C98	0.8707 (5)	0.8330 (3)	0.1544 (2)	0.063 (2)	
H98A	0.8592	0.8334	0.1835	0.076*	
H98B	0.8885	0.8716	0.1467	0.076*	
C99	0.8341 (4)	0.6512 (3)	0.0950 (2)	0.0473 (17)	
H99A	0.8376	0.6760	0.1190	0.057*	
H99B	0.7818	0.6333	0.0949	0.057*	

### Atomic displacement parameters (Å^2^)

**Table:**

	*U*^11^	*U*^22^	*U*^33^	*U*^12^	*U*^13^	*U*^23^
Ru	0.01239 (18)	0.01262 (18)	0.01425 (18)	0.00034 (15)	0.00010 (16)	−0.00034 (16)
Cl1	0.0448 (8)	0.0227 (6)	0.0181 (6)	0.0103 (6)	0.0046 (6)	0.0013 (5)
Cl2	0.0485 (12)	0.0664 (14)	0.145 (2)	−0.0060 (10)	0.0052 (13)	0.0341 (15)
Cl3	0.0603 (12)	0.0550 (12)	0.0967 (16)	0.0017 (10)	−0.0156 (11)	−0.0141 (11)
Cl4	0.0636 (12)	0.0825 (14)	0.0327 (9)	−0.0088 (10)	0.0018 (8)	0.0111 (9)
Cl5	0.0471 (11)	0.0521 (12)	0.134 (2)	−0.0146 (9)	−0.0125 (12)	0.0166 (13)
S	0.0284 (7)	0.0234 (7)	0.0216 (7)	0.0031 (6)	−0.0011 (6)	0.0010 (5)
P1	0.0153 (6)	0.0139 (6)	0.0170 (6)	0.0013 (5)	0.0003 (5)	−0.0001 (5)
P2	0.0133 (6)	0.0155 (6)	0.0159 (6)	0.0002 (5)	−0.0003 (5)	−0.0014 (5)
P3	0.0129 (6)	0.0147 (6)	0.0162 (6)	−0.0003 (5)	−0.0004 (5)	0.0005 (5)
P4	0.0165 (6)	0.0144 (6)	0.0155 (6)	0.0009 (5)	0.0003 (5)	−0.0008 (5)
F1	0.066 (3)	0.052 (2)	0.035 (2)	0.023 (2)	0.0147 (18)	−0.0037 (18)
F2	0.080 (3)	0.0223 (17)	0.039 (2)	0.0076 (17)	−0.0109 (19)	0.0042 (15)
F3	0.064 (2)	0.035 (2)	0.043 (2)	0.0024 (18)	−0.0238 (19)	−0.0094 (17)
O1	0.0193 (17)	0.0143 (17)	0.0209 (18)	−0.0018 (14)	−0.0012 (14)	−0.0015 (15)
O2	0.0187 (17)	0.0162 (17)	0.0156 (17)	0.0006 (14)	0.0030 (13)	−0.0054 (14)
O3	0.0186 (17)	0.0163 (17)	0.0149 (17)	0.0014 (14)	−0.0003 (14)	−0.0008 (14)
O4	0.0288 (19)	0.0152 (17)	0.0152 (17)	0.0007 (15)	0.0001 (14)	−0.0007 (14)
O5	0.036 (2)	0.0234 (19)	0.0202 (18)	0.0046 (17)	0.0038 (17)	0.0055 (15)
O6	0.026 (2)	0.048 (3)	0.030 (2)	0.0046 (18)	0.0058 (17)	0.0086 (19)
O7	0.0201 (18)	0.028 (2)	0.027 (2)	0.0027 (15)	0.0000 (15)	−0.0016 (16)
C1	0.016 (2)	0.022 (3)	0.019 (2)	−0.001 (2)	0.002 (2)	−0.002 (2)
C2	0.025 (3)	0.017 (3)	0.022 (3)	0.001 (2)	0.000 (2)	−0.006 (2)
C3	0.026 (3)	0.022 (3)	0.031 (3)	0.002 (2)	0.004 (2)	−0.012 (2)
C4	0.026 (3)	0.026 (3)	0.029 (3)	−0.002 (2)	0.002 (2)	−0.012 (2)
C5	0.018 (3)	0.027 (3)	0.022 (3)	−0.002 (2)	−0.003 (2)	−0.004 (2)
C6	0.019 (2)	0.018 (3)	0.019 (3)	0.0000 (19)	0.004 (2)	−0.004 (2)
C11	0.020 (3)	0.015 (2)	0.022 (3)	0.000 (2)	0.001 (2)	0.005 (2)
C12	0.030 (3)	0.034 (3)	0.019 (3)	0.004 (2)	0.007 (2)	0.008 (2)
C13	0.040 (3)	0.028 (3)	0.023 (3)	−0.001 (3)	0.001 (2)	0.010 (2)
C14	0.034 (3)	0.020 (3)	0.030 (3)	0.002 (2)	−0.007 (2)	0.008 (2)
C15	0.025 (3)	0.016 (3)	0.024 (3)	0.002 (2)	−0.003 (2)	0.006 (2)
C16	0.020 (3)	0.020 (3)	0.018 (3)	−0.002 (2)	−0.001 (2)	0.003 (2)
C21	0.014 (2)	0.017 (2)	0.015 (2)	0.0027 (19)	−0.0020 (19)	0.004 (2)
C22	0.020 (3)	0.018 (2)	0.026 (3)	0.002 (2)	−0.002 (2)	0.001 (2)
C23	0.020 (3)	0.023 (3)	0.030 (3)	0.008 (2)	0.001 (2)	0.003 (2)
C24	0.017 (3)	0.022 (3)	0.031 (3)	0.001 (2)	−0.002 (2)	0.002 (2)
C25	0.024 (3)	0.017 (3)	0.027 (3)	−0.005 (2)	−0.007 (2)	−0.004 (2)
C26	0.022 (3)	0.018 (3)	0.020 (3)	0.005 (2)	−0.004 (2)	0.001 (2)
C31	0.018 (3)	0.013 (2)	0.023 (3)	0.005 (2)	0.004 (2)	0.003 (2)
C32	0.022 (3)	0.018 (3)	0.028 (3)	0.001 (2)	0.002 (2)	0.004 (2)
C33	0.031 (3)	0.016 (3)	0.043 (4)	0.000 (2)	0.012 (3)	0.001 (2)
C34	0.033 (3)	0.021 (3)	0.041 (4)	0.009 (2)	0.019 (3)	0.014 (3)
C35	0.028 (3)	0.029 (3)	0.026 (3)	0.015 (2)	0.005 (2)	0.004 (2)
C36	0.019 (3)	0.021 (3)	0.021 (3)	0.003 (2)	0.002 (2)	0.005 (2)
C41	0.013 (2)	0.016 (2)	0.025 (3)	0.0026 (19)	0.000 (2)	0.001 (2)
C42	0.016 (2)	0.022 (3)	0.027 (3)	0.001 (2)	0.000 (2)	−0.002 (2)
C43	0.017 (3)	0.022 (3)	0.033 (3)	−0.004 (2)	−0.002 (2)	−0.005 (2)
C44	0.016 (3)	0.025 (3)	0.041 (3)	−0.005 (2)	0.002 (2)	−0.002 (2)
C45	0.022 (3)	0.023 (3)	0.028 (3)	0.002 (2)	0.009 (2)	0.003 (2)
C46	0.017 (2)	0.018 (3)	0.020 (3)	0.001 (2)	0.000 (2)	−0.002 (2)
C51	0.015 (2)	0.018 (2)	0.019 (3)	−0.0018 (19)	−0.007 (2)	−0.001 (2)
C52	0.023 (3)	0.018 (3)	0.020 (3)	−0.007 (2)	−0.003 (2)	0.000 (2)
C53	0.037 (3)	0.025 (3)	0.022 (3)	−0.008 (2)	−0.010 (2)	0.002 (2)
C54	0.034 (3)	0.023 (3)	0.033 (3)	0.003 (2)	−0.019 (3)	0.001 (2)
C55	0.025 (3)	0.024 (3)	0.036 (3)	0.006 (2)	−0.010 (2)	−0.006 (3)
C56	0.019 (3)	0.023 (3)	0.023 (3)	0.001 (2)	−0.005 (2)	−0.004 (2)
C61	0.014 (2)	0.012 (2)	0.023 (3)	−0.0037 (19)	0.0052 (19)	0.001 (2)
C62	0.021 (3)	0.018 (3)	0.023 (3)	−0.002 (2)	0.004 (2)	−0.002 (2)
C63	0.016 (3)	0.024 (3)	0.035 (3)	0.003 (2)	0.007 (2)	0.002 (2)
C64	0.026 (3)	0.028 (3)	0.034 (3)	0.002 (2)	0.010 (2)	−0.008 (3)
C65	0.031 (3)	0.025 (3)	0.025 (3)	−0.007 (2)	0.004 (2)	−0.006 (2)
C66	0.018 (3)	0.024 (3)	0.024 (3)	−0.001 (2)	0.002 (2)	−0.003 (2)
C71	0.017 (2)	0.012 (2)	0.024 (3)	0.0013 (19)	−0.003 (2)	0.001 (2)
C72	0.017 (2)	0.024 (3)	0.023 (3)	−0.004 (2)	0.000 (2)	−0.004 (2)
C73	0.021 (3)	0.027 (3)	0.038 (3)	−0.005 (2)	0.004 (2)	0.006 (2)
C74	0.020 (3)	0.027 (3)	0.040 (3)	−0.003 (2)	−0.007 (3)	0.000 (3)
C75	0.025 (3)	0.027 (3)	0.024 (3)	0.000 (2)	−0.007 (2)	−0.005 (2)
C76	0.017 (2)	0.021 (3)	0.025 (3)	0.004 (2)	−0.001 (2)	0.001 (2)
C81	0.016 (2)	0.022 (3)	0.021 (3)	0.003 (2)	0.000 (2)	−0.007 (2)
C82	0.020 (3)	0.025 (3)	0.022 (3)	0.000 (2)	−0.001 (2)	−0.003 (2)
C83	0.028 (3)	0.031 (3)	0.025 (3)	0.000 (2)	0.005 (2)	−0.007 (2)
C84	0.020 (3)	0.047 (4)	0.040 (4)	0.004 (3)	−0.002 (3)	−0.015 (3)
C85	0.030 (3)	0.034 (3)	0.040 (4)	0.016 (3)	−0.005 (3)	−0.005 (3)
C86	0.024 (3)	0.023 (3)	0.037 (3)	0.006 (2)	−0.002 (2)	−0.007 (2)
C91	0.017 (2)	0.015 (2)	0.020 (3)	0.0049 (19)	−0.004 (2)	−0.004 (2)
C92	0.024 (3)	0.017 (3)	0.023 (3)	−0.002 (2)	0.000 (2)	0.000 (2)
C93	0.025 (3)	0.020 (3)	0.033 (3)	−0.001 (2)	−0.005 (2)	−0.005 (2)
C94	0.026 (3)	0.020 (3)	0.028 (3)	0.005 (2)	−0.014 (2)	−0.007 (2)
C95	0.021 (3)	0.028 (3)	0.016 (2)	0.010 (2)	−0.001 (2)	−0.007 (2)
C96	0.019 (3)	0.019 (3)	0.017 (3)	0.004 (2)	−0.003 (2)	−0.003 (2)
C97	0.054 (4)	0.033 (3)	0.024 (3)	0.005 (3)	−0.004 (3)	0.004 (3)
C98	0.086 (6)	0.057 (5)	0.045 (4)	0.020 (4)	0.002 (4)	0.006 (4)
C99	0.052 (4)	0.049 (4)	0.042 (4)	−0.019 (3)	−0.005 (3)	0.008 (3)

### Geometric parameters (Å, °)

**Table:**

Ru—P2	2.2237 (13)	C32—H32	0.9300
Ru—P3	2.2430 (13)	C33—C34	1.387 (8)
Ru—Cl1	2.3838 (13)	C33—H33	0.9300
Ru—P1	2.3935 (12)	C34—C35	1.383 (8)
Ru—P4	2.4170 (13)	C34—H34	0.9300
Cl2—C98	1.751 (8)	C35—C36	1.388 (7)
Cl3—C98	1.757 (7)	C35—H35	0.9300
Cl4—C99	1.753 (6)	C36—H36	0.9300
Cl5—C99	1.743 (7)	C41—C46	1.395 (7)
S—O5	1.438 (4)	C41—C42	1.404 (7)
S—O7	1.439 (4)	C42—C43	1.381 (7)
S—O6	1.448 (4)	C42—H42	0.9300
S—C97	1.809 (6)	C43—C44	1.380 (7)
P1—O1	1.611 (3)	C43—H43	0.9300
P1—C21	1.821 (5)	C44—C45	1.381 (7)
P1—C31	1.835 (5)	C44—H44	0.9300
P2—O2	1.613 (3)	C45—C46	1.397 (7)
P2—C51	1.822 (5)	C45—H45	0.9300
P2—C41	1.823 (5)	C46—H46	0.9300
P3—O3	1.612 (3)	C51—C56	1.389 (7)
P3—C71	1.820 (5)	C51—C52	1.410 (7)
P3—C61	1.832 (5)	C52—C53	1.379 (7)
P4—O4	1.611 (3)	C52—H52	0.9300
P4—C81	1.819 (5)	C53—C54	1.383 (8)
P4—C91	1.834 (5)	C53—H53	0.9300
F1—C97	1.350 (7)	C54—C55	1.382 (8)
F2—C97	1.332 (6)	C54—H54	0.9300
F3—C97	1.333 (7)	C55—C56	1.379 (7)
O1—C1	1.457 (6)	C55—H55	0.9300
O2—C6	1.447 (6)	C56—H56	0.9300
O3—C11	1.451 (6)	C61—C62	1.385 (7)
O4—C16	1.430 (6)	C61—C66	1.395 (7)
C1—C6	1.505 (7)	C62—C63	1.389 (7)
C1—C2	1.520 (7)	C62—H62	0.9300
C1—H1	0.9800	C63—C64	1.380 (8)
C2—C3	1.526 (7)	C63—H63	0.9300
C2—H2A	0.9700	C64—C65	1.376 (7)
C2—H2B	0.9700	C64—H64	0.9300
C3—C4	1.519 (7)	C65—C66	1.391 (7)
C3—H3A	0.9700	C65—H65	0.9300
C3—H3B	0.9700	C66—H66	0.9300
C4—C5	1.528 (7)	C71—C76	1.400 (7)
C4—H4A	0.9700	C71—C72	1.402 (7)
C4—H4B	0.9700	C72—C73	1.373 (7)
C5—C6	1.533 (7)	C72—H72	0.9300
C5—H5A	0.9700	C73—C74	1.386 (8)
C5—H5B	0.9700	C73—H73	0.9300
C6—H6	0.9800	C74—C75	1.370 (7)
C11—C16	1.517 (7)	C74—H74	0.9300
C11—C12	1.518 (7)	C75—C76	1.391 (7)
C11—H11	0.9800	C75—H75	0.9300
C12—C13	1.523 (7)	C76—H76	0.9300
C12—H12A	0.9700	C81—C82	1.392 (7)
C12—H12B	0.9700	C81—C86	1.394 (7)
C13—C14	1.523 (8)	C82—C83	1.388 (7)
C13—H13A	0.9700	C82—H82	0.9300
C13—H13B	0.9700	C83—C84	1.380 (8)
C14—C15	1.521 (7)	C83—H83	0.9300
C14—H14A	0.9700	C84—C85	1.381 (8)
C14—H14B	0.9700	C84—H84	0.9300
C15—C16	1.527 (7)	C85—C86	1.388 (8)
C15—H15A	0.9700	C85—H85	0.9300
C15—H15B	0.9700	C86—H86	0.9300
C16—H16	0.9800	C91—C96	1.392 (7)
C21—C22	1.390 (6)	C91—C92	1.403 (7)
C21—C26	1.396 (7)	C92—C93	1.381 (7)
C22—C23	1.385 (7)	C92—H92	0.9300
C22—H22	0.9300	C93—C94	1.368 (7)
C23—C24	1.386 (7)	C93—H93	0.9300
C23—H23	0.9300	C94—C95	1.395 (7)
C24—C25	1.378 (7)	C94—H94	0.9300
C24—H24	0.9300	C95—C96	1.379 (7)
C25—C26	1.388 (7)	C95—H95	0.9300
C25—H25	0.9300	C96—H96	0.9300
C26—H26	0.9300	C98—H98A	0.9700
C31—C32	1.396 (7)	C98—H98B	0.9700
C31—C36	1.396 (7)	C99—H99A	0.9700
C32—C33	1.390 (7)	C99—H99B	0.9700
			
P2—Ru—P3	87.81 (5)	C31—C32—H32	119.9
P2—Ru—Cl1	131.42 (5)	C34—C33—C32	120.2 (5)
P3—Ru—Cl1	140.73 (5)	C34—C33—H33	119.9
P2—Ru—P1	89.44 (4)	C32—C33—H33	119.9
P3—Ru—P1	99.68 (4)	C35—C34—C33	120.0 (5)
Cl1—Ru—P1	84.49 (4)	C35—C34—H34	120.0
P2—Ru—P4	99.48 (4)	C33—C34—H34	120.0
P3—Ru—P4	89.39 (4)	C34—C35—C36	120.1 (5)
Cl1—Ru—P4	83.10 (4)	C34—C35—H35	119.9
P1—Ru—P4	167.55 (4)	C36—C35—H35	119.9
O5—S—O7	115.2 (2)	C35—C36—C31	120.4 (5)
O5—S—O6	115.4 (2)	C35—C36—H36	119.8
O7—S—O6	114.6 (2)	C31—C36—H36	119.8
O5—S—C97	103.8 (2)	C46—C41—C42	119.2 (4)
O7—S—C97	104.2 (3)	C46—C41—P2	123.1 (4)
O6—S—C97	101.1 (3)	C42—C41—P2	117.7 (4)
O1—P1—C21	106.7 (2)	C43—C42—C41	120.1 (5)
O1—P1—C31	95.4 (2)	C43—C42—H42	119.9
C21—P1—C31	99.6 (2)	C41—C42—H42	119.9
O1—P1—Ru	120.89 (13)	C44—C43—C42	120.3 (5)
C21—P1—Ru	112.07 (16)	C44—C43—H43	119.9
C31—P1—Ru	119.11 (15)	C42—C43—H43	119.9
O2—P2—C51	97.2 (2)	C43—C44—C45	120.5 (5)
O2—P2—C41	105.1 (2)	C43—C44—H44	119.7
C51—P2—C41	100.0 (2)	C45—C44—H44	119.7
O2—P2—Ru	115.97 (13)	C44—C45—C46	119.9 (5)
C51—P2—Ru	122.52 (16)	C44—C45—H45	120.1
C41—P2—Ru	113.23 (17)	C46—C45—H45	120.1
O3—P3—C71	106.5 (2)	C41—C46—C45	120.0 (5)
O3—P3—C61	97.1 (2)	C41—C46—H46	120.0
C71—P3—C61	98.6 (2)	C45—C46—H46	120.0
O3—P3—Ru	114.53 (13)	C56—C51—C52	118.0 (4)
C71—P3—Ru	110.89 (17)	C56—C51—P2	121.3 (4)
C61—P3—Ru	126.64 (15)	C52—C51—P2	120.6 (4)
O4—P4—C81	104.2 (2)	C53—C52—C51	120.3 (5)
O4—P4—C91	93.7 (2)	C53—C52—H52	119.8
C81—P4—C91	100.8 (2)	C51—C52—H52	119.8
O4—P4—Ru	123.02 (13)	C52—C53—C54	120.7 (5)
C81—P4—Ru	113.65 (17)	C52—C53—H53	119.7
C91—P4—Ru	117.66 (15)	C54—C53—H53	119.7
C1—O1—P1	130.4 (3)	C55—C54—C53	119.5 (5)
C6—O2—P2	130.8 (3)	C55—C54—H54	120.3
C11—O3—P3	129.7 (3)	C53—C54—H54	120.3
C16—O4—P4	136.0 (3)	C56—C55—C54	120.3 (5)
O1—C1—C6	108.9 (4)	C56—C55—H55	119.8
O1—C1—C2	106.1 (4)	C54—C55—H55	119.8
C6—C1—C2	109.6 (4)	C55—C56—C51	121.2 (5)
O1—C1—H1	110.7	C55—C56—H56	119.4
C6—C1—H1	110.7	C51—C56—H56	119.4
C2—C1—H1	110.7	C62—C61—C66	119.0 (4)
C1—C2—C3	110.1 (4)	C62—C61—P3	120.5 (4)
C1—C2—H2A	109.6	C66—C61—P3	120.4 (4)
C3—C2—H2A	109.6	C61—C62—C63	120.6 (5)
C1—C2—H2B	109.6	C61—C62—H62	119.7
C3—C2—H2B	109.6	C63—C62—H62	119.7
H2A—C2—H2B	108.2	C64—C63—C62	120.0 (5)
C4—C3—C2	111.2 (4)	C64—C63—H63	120.0
C4—C3—H3A	109.4	C62—C63—H63	120.0
C2—C3—H3A	109.4	C65—C64—C63	120.1 (5)
C4—C3—H3B	109.4	C65—C64—H64	120.0
C2—C3—H3B	109.4	C63—C64—H64	120.0
H3A—C3—H3B	108.0	C64—C65—C66	120.2 (5)
C3—C4—C5	111.4 (4)	C64—C65—H65	119.9
C3—C4—H4A	109.4	C66—C65—H65	119.9
C5—C4—H4A	109.4	C65—C66—C61	120.1 (5)
C3—C4—H4B	109.4	C65—C66—H66	120.0
C5—C4—H4B	109.4	C61—C66—H66	120.0
H4A—C4—H4B	108.0	C76—C71—C72	119.1 (4)
C4—C5—C6	112.9 (4)	C76—C71—P3	122.6 (4)
C4—C5—H5A	109.0	C72—C71—P3	118.3 (4)
C6—C5—H5A	109.0	C73—C72—C71	120.4 (5)
C4—C5—H5B	109.0	C73—C72—H72	119.8
C6—C5—H5B	109.0	C71—C72—H72	119.8
H5A—C5—H5B	107.8	C72—C73—C74	119.8 (5)
O2—C6—C1	111.6 (4)	C72—C73—H73	120.1
O2—C6—C5	104.4 (4)	C74—C73—H73	120.1
C1—C6—C5	112.0 (4)	C75—C74—C73	120.7 (5)
O2—C6—H6	109.6	C75—C74—H74	119.6
C1—C6—H6	109.6	C73—C74—H74	119.6
C5—C6—H6	109.6	C74—C75—C76	120.2 (5)
O3—C11—C16	109.3 (4)	C74—C75—H75	119.9
O3—C11—C12	106.9 (4)	C76—C75—H75	119.9
C16—C11—C12	112.9 (4)	C75—C76—C71	119.7 (5)
O3—C11—H11	109.2	C75—C76—H76	120.2
C16—C11—H11	109.2	C71—C76—H76	120.2
C12—C11—H11	109.2	C82—C81—C86	119.3 (5)
C11—C12—C13	109.9 (4)	C82—C81—P4	121.1 (4)
C11—C12—H12A	109.7	C86—C81—P4	119.6 (4)
C13—C12—H12A	109.7	C83—C82—C81	120.5 (5)
C11—C12—H12B	109.7	C83—C82—H82	119.7
C13—C12—H12B	109.7	C81—C82—H82	119.7
H12A—C12—H12B	108.2	C84—C83—C82	119.6 (5)
C14—C13—C12	110.3 (4)	C84—C83—H83	120.2
C14—C13—H13A	109.6	C82—C83—H83	120.2
C12—C13—H13A	109.6	C83—C84—C85	120.5 (5)
C14—C13—H13B	109.6	C83—C84—H84	119.7
C12—C13—H13B	109.6	C85—C84—H84	119.7
H13A—C13—H13B	108.1	C84—C85—C86	120.2 (5)
C15—C14—C13	110.8 (4)	C84—C85—H85	119.9
C15—C14—H14A	109.5	C86—C85—H85	119.9
C13—C14—H14A	109.5	C85—C86—C81	119.9 (5)
C15—C14—H14B	109.5	C85—C86—H86	120.0
C13—C14—H14B	109.5	C81—C86—H86	120.0
H14A—C14—H14B	108.1	C96—C91—C92	118.3 (4)
C14—C15—C16	112.0 (4)	C96—C91—P4	121.8 (4)
C14—C15—H15A	109.2	C92—C91—P4	119.7 (4)
C16—C15—H15A	109.2	C93—C92—C91	120.8 (5)
C14—C15—H15B	109.2	C93—C92—H92	119.6
C16—C15—H15B	109.2	C91—C92—H92	119.6
H15A—C15—H15B	107.9	C94—C93—C92	120.1 (5)
O4—C16—C11	109.2 (4)	C94—C93—H93	119.9
O4—C16—C15	105.5 (4)	C92—C93—H93	119.9
C11—C16—C15	109.7 (4)	C93—C94—C95	120.0 (5)
O4—C16—H16	110.8	C93—C94—H94	120.0
C11—C16—H16	110.8	C95—C94—H94	120.0
C15—C16—H16	110.8	C96—C95—C94	120.2 (5)
C22—C21—C26	119.5 (4)	C96—C95—H95	119.9
C22—C21—P1	121.0 (4)	C94—C95—H95	119.9
C26—C21—P1	119.5 (4)	C95—C96—C91	120.5 (5)
C23—C22—C21	119.8 (5)	C95—C96—H96	119.8
C23—C22—H22	120.1	C91—C96—H96	119.8
C21—C22—H22	120.1	F2—C97—F3	107.9 (5)
C22—C23—C24	120.7 (5)	F2—C97—F1	106.7 (5)
C22—C23—H23	119.7	F3—C97—F1	107.2 (5)
C24—C23—H23	119.7	F2—C97—S	112.0 (4)
C25—C24—C23	119.6 (5)	F3—C97—S	111.8 (4)
C25—C24—H24	120.2	F1—C97—S	111.0 (4)
C23—C24—H24	120.2	Cl2—C98—Cl3	111.9 (4)
C24—C25—C26	120.5 (5)	Cl2—C98—H98A	109.2
C24—C25—H25	119.7	Cl3—C98—H98A	109.2
C26—C25—H25	119.7	Cl2—C98—H98B	109.2
C25—C26—C21	119.9 (4)	Cl3—C98—H98B	109.2
C25—C26—H26	120.0	H98A—C98—H98B	107.9
C21—C26—H26	120.0	Cl5—C99—Cl4	112.6 (4)
C32—C31—C36	119.0 (4)	Cl5—C99—H99A	109.1
C32—C31—P1	120.2 (4)	Cl4—C99—H99A	109.1
C36—C31—P1	120.8 (4)	Cl5—C99—H99B	109.1
C33—C32—C31	120.2 (5)	Cl4—C99—H99B	109.1
C33—C32—H32	119.9	H99A—C99—H99B	107.8

## References

[bb1] Allen, F. H. (2002). *Acta Cryst.* B**58**, 380–388. 10.1107/s0108768102003890 12037359

[bb2] Au-Yeung, T. T.-L. & Chan, A. S. C. (2004). *Coord. Chem. Rev.* **248**, 2151–2164.

[bb3] Burnett, M. N. & Johnson, C. K. (1996). *ORTEPIII* Report ORNL-6895. Oak Ridge National Laboratory, Tennessee, USA.

[bb4] Clark, G. R., Falshaw, A., Gainsford, G. J., Lensink, C., Slade, A. T. & Wright, L. J. (2009). *Polyhedron, *Submitted.

[bb5] Falshaw, A., Gainsford, G. J., Lensink, C., Slade, A. T. & Wright, L. J. (2007). *Polyhedron*, **26**, 329–337.

[bb6] Knowles, W. S. & Noyori, R. (2007). *Acc. Chem. Res.* **40**, 1238–1239. 10.1021/ar7000809 18085747

[bb7] Sheldrick, G. M. (1996). *SADABS* University of Göttingen, Germany.

[bb8] Sheldrick, G. M. (2008). *Acta Cryst.* A**64**, 112–122. 10.1107/S0108767307043930 18156677

[bb9] Siemens (1995). *SMART* and *SAINT* Siemens Analytical X-ray Instruments Inc., Madison, Wisconsin, USA.

[bb10] Zhang, X. (2004). *Tetrahedron Asymmetry*, **15**, 2099–2100.

[bb11] Zhang, W., Chi, Y. & Zhang, X. (2007). *Acc. Chem. Res.* **40**, 1278–1290. 10.1021/ar7000028 17506517

